# A High-Speed Acoustic Echo Canceller Based on Grey Wolf Optimization and Particle Swarm Optimization Algorithms

**DOI:** 10.3390/biomimetics9070381

**Published:** 2024-06-23

**Authors:** Eduardo Pichardo, Juan G. Avalos, Giovanny Sánchez, Eduardo Vazquez, Linda K. Toscano

**Affiliations:** 1 Tecnologico de Monterrey, School of Engineering and Sciences, Calle del Puente 222, Col. Ejidos de Huipulco Tlalpan, Ciudad de Mexico 14380, Mexico; epichardom@tec.mx; 2 Instituto Politécnico Nacional, ESIME Culhuacan, Av. Santa Ana No. 1000, Ciudad de Mexico 04260, Mexico; edvazquezf@ipn.mx (E.V.); ltoscano@ipn.mx (L.K.T.)

**Keywords:** grey wolf optimization, particle swarm optimization, acoustic echo canceller, adaptive filtering

## Abstract

Currently, the use of acoustic echo cancellers (AECs) plays a crucial role in IoT applications, such as voice control appliances, hands-free telephony and intelligent voice control devices, among others. Therefore, these IoT devices are mostly controlled by voice commands. However, the performance of these devices is significantly affected by echo noise in real acoustic environments. Despite good results being achieved in terms of echo noise reductions using conventional adaptive filtering based on gradient optimization algorithms, recently, the use of bio-inspired algorithms has attracted significant attention in the science community, since these algorithms exhibit a faster convergence rate when compared with gradient optimization algorithms. To date, several authors have tried to develop high-performance AEC systems to offer high-quality and realistic sound. In this work, we present a new AEC system based on the grey wolf optimization (GWO) and particle swarm optimization (PSO) algorithms to guarantee a higher convergence speed compared with previously reported solutions. This improvement potentially allows for high tracking capabilities. This aspect has special relevance in real acoustic environments since it indicates the rate at which noise is reduced.

## 1. Introduction

Nowadays, acoustic echo cancellation (AEC) is considered as an important area of speech enhancement and its improvement is still a important issue in audio communication. In particular, its impact is growing considerably, since its use is highly demanded in consumer IoT devices controlled by voice commands. Recently, new schemes have been developed to overcome the performance problems of conventional adaptive filtering structures [1,2]. To ensure a high speech quality and speech intelligibility, cutting-edge metaheuristic algorithms have emerged and are considered as potential solutions, since conventional step-descent adaptive filtering algorithms offer limited performance. Recent studies have proven that the use of metaheuristic algorithms has increased the performance of advanced filtering applications, such as active noise control (ANC) [3,4,5,6,7,8,9,10,11,12,13,14], enhancement of speech or suppression of noise [15,16,17] and acoustic echo cancellation. Regarding the latter application, Diana et al. [18] proposed a hybrid metaheuristic technique based on the artificial bee colony (ABC) and the Kernel Adaptive Improved Proportionate and Normalized Least Mean Square (KIPNLMS) algorithm. The authors used these two algorithms to achieve a high echo cancellation efficiency under linear and nonlinear distortions of the speech signal. On the other hand, Kimoto et al. [19] proposed a multichannel AEC system based on a variant of the particle swarm optimization (PSO) algorithm called deterministic particle swarm optimization (D-PSO). Merabti et al. [20] presented an AEC system based on a genetic algorithm (GA) to estimate the room impulse response and nonlinear function parameters. Recently, Anides et al. [21] proposed an efficient processor to simulate a new variant of the PSO algorithm based on the Markovian switching technique. On the other hand, Pichardo et al. [22] used grey wolf optimization and the LMS algorithm to increase the convergence speed.

Analyzing previous works, two large challenges remain in the development of efficient AEC systems to significantly reduce the echo noise:

- The first challenge is linked to the achievement of a high convergence speed and good tracking capabilities in AEC systems.
- The second challenge lies in improving the extremely time-consuming process in the design of these metaheuristic algorithms, since most of them require a trial-and-error process for parameter setting.

Here, we present two contributions to overcome the above challenges:

1. We propose a new AEC system that uses convex combination based on the PSO algorithm and the GWO algorithm to achieve a better echo cancellation performance. Here, the use of GWO allows us to achieve a fast convergence rate, while the use of the PSO algorithm allows us to obtain a high exploitation performance by adjusting inertia weights and acceleration coefficients. The results show that our proposed method outperforms existing approaches in terms of the ERLE level and tracking capabilities to significantly reduce the background noise, reverberation and other types of interference.
2. To improve the time-consuming process, we use the GWO algorithm, since this requires fewer parameters to be tuned. In addition, we carefully select another metaheuristic algorithm to result in algorithms with complementary capabilities. As a consequence, we can reduce parameter tuning, which is beneficial, especially when used in practical applications.

In general terms, the study of new metaheuristic algorithms opens new horizons in the development of high-performance AEC systems. Specifically, the use of metaheuristic algorithms in the development of AEC systems allows us to achieve higher convergence speeds and offer higher tracking capabilities in comparison with gradient descent adaptive filtering algorithms.

## 2. The Proposed Convex GWO/PSO Algorithm

Recently, the development of combination schemes has attracted significant attention, since the outputs of several filters are mixed together to obtain an overall output of increased quality. To achieve this, several authors use a linear combination of these filters. In this work, we use the GWO algorithm because of its simplicity and low number of parameters that need to be adjusted [23]. However, one of its critical limitations appears when dealing with a large number of variables and local solutions. Specifically, its accelerated exploitation and fast convergence speed can be seen as disadvantages since they cause it to fall into local solutions easily. On the other hand, the particle swarm optimization (PSO) algorithm maintains a balance between exploration and exploitation by adjusting certain parameters. Therefore, the algorithm exhibits better exploration or exploitation performances according to the requirements. This features allows us to obtain a flexible and effective algorithm. Here, we use the GWO algorithm to achieve a high exploitation performance and PSO is used to obtain a high exploration performance by adjusting inertia weights and acceleration coefficients.

### 2.1. GWO Algorithm

The GWO algorithm is a population-based metaheuristic algorithm that intends to mimic the social behavior and hunting strategy of grey wolves in nature [24]. This algorithm employs four types of grey wolves to represent the leadership hierarchy of a wolf pack: alpha (α), which represents the fittest solution; omega (ω), which are the search agents; and finally, beta (β) and delta (δ), which are the second and third best solutions, respectively. To simulate the GWO algorithm, the following equations are used:

(1) $$\vec{W}\left(n+1\right)={\vec{W}}_{p}\left(n\right)-\vec{A}\cdot |\vec{C}\cdot {\vec{W}}_{p}\left(n\right)-\vec{W}\left(n\right)|$$

where *n* indicates the current iteration, $\vec{W}$ represents the position of a grey wolf, ${\vec{W}}_{p}$ is the position vector of the prey, and $\vec{A}$ and $\vec{C}$ denote coefficient vectors, which are calculated by

(2) $$\vec{A}=2\vec{a}\cdot {\vec{r}}_{1}-\vec{a}$$

(3) $$\vec{C}=2{\vec{r}}_{2}$$

The parameters ${\vec{r}}_{1}$ and ${\vec{r}}_{2}$ represent random vectors in [0,1] and $\vec{a}$ is the tuning parameter for exploration and exploitation, which is linearly decreased from 2 to 0 and is calculated using:

(4) $$\vec{a}\left(n\right)=2-\frac{2n}{MaxIter}$$

where MaxIter is the total number of iterations. The updated positions of the search agents are expressed by

(5) $${\vec{W}}_{1}\left(n\right)={\vec{W}}_{\alpha }\left(n\right)-{\vec{A}}_{1}\cdot |{\vec{C}}_{1}\cdot {\vec{W}}_{\alpha }\left(n\right)-\vec{W}\left(n\right)|$$

(6) $${\vec{W}}_{2}\left(n\right)={\vec{W}}_{\beta }\left(n\right)-{\vec{A}}_{2}\cdot |{\vec{C}}_{2}\cdot {\vec{W}}_{\beta }\left(n\right)-\vec{W}\left(n\right)|$$

(7) $${\vec{W}}_{3}\left(n\right)={\vec{W}}_{\delta }\left(n\right)-{\vec{A}}_{3}\cdot |{\vec{C}}_{3}\cdot {\vec{W}}_{\delta }\left(n\right)-\vec{W}\left(n\right)|$$

(8) $${\vec{W}}_{p}\left(n+1\right)=\frac{{\vec{W}}_{1}\left(n\right)+{\vec{W}}_{2}\left(n\right)+{\vec{W}}_{3}\left(n\right)}{3}$$

The best three wolves at each iteration are defined by ${\vec{W}}_{\alpha }\left(n\right),{\vec{W}}_{\beta }\left(n\right)$ and ${\vec{W}}_{\delta }\left(n\right)$, and ${\vec{W}}_{p}\left(n+1\right)$ is the new position of the prey.

### 2.2. PSO Algorithm

Particle swarm optimization (PSO) is a bio-inspired algorithm that mimics the collective behavior of a swarm of particles to find an optimal solution [25]. Each particle has a unique position and velocity, which are updated based on the individual best position and the best position of the swarm. After calculating the velocity of each particle, the new velocity is applied to the particle’s previous position to update its position. The equation for updating the velocity and position of each particle is as follows:

(9) $$v_{i}\left(n+1\right)=\phi \cdot v_{i}\left(n\right)+c_{1}\cdot r_{1}\left[x_{pbest}-x_{i}\left(n\right)\right]+c_{2}\cdot r_{2}\left[x_{gbest}-x_{i}\left(n\right)\right]$$

(10) $$x_{i}\left(n+1\right)=x_{i}\left(n\right)+v_{i}\left(n+1\right)$$

where $v_{i}\left(n\right)$ is the velocity of particle *i* at time *n*, ϕ is the inertia weight, $c_{1}$ and $c_{2}$ are acceleration constants, $r_{1}$ and $r_{2}$ are random numbers between 0 and 1, $x_{pbest}$ is the best position of particle *i*, $x_{i}\left(n\right)$ is the current position of particle *i*, and $x_{gbest}$ is the global best position of the swarm.

### 2.3. Convex GWO/PSO Algorithm

To date, several works have proven that combining two adaptive algorithms has several advantages; one of them is linked to the improvement in the performance. In this work, we present a new algorithm that combines two swarm intelligence techniques (the GWO and PSO algorithms) to improve tracking capabilities and to ensure a high convergence rate.

**Figure 1:**
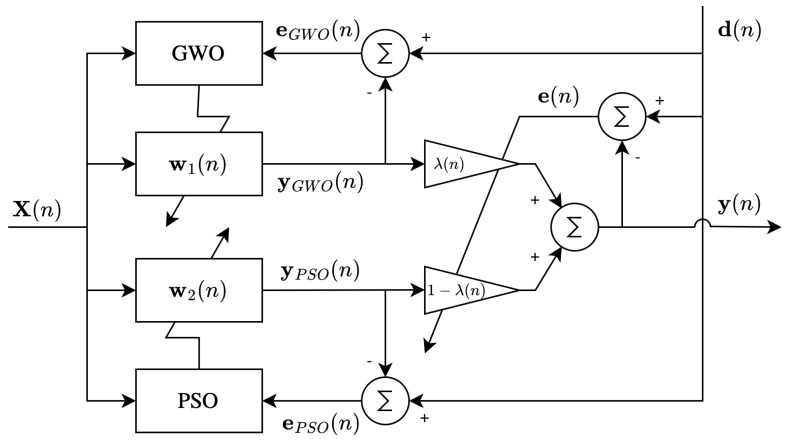
The proposed structure using the GWO/PSO algorithm.

In the proposed structure (see Figure 1), the input signal, X(n), is defined by:

(11) x(n)=[x(nL),x(nL−1),⋯x(nL−N+1)]

(12) $$X\left(n\right)=\left[\begin{array}{cccc} x(nL) & x(nL-1) & \cdots & x(nL-N+1)\\ x(nL-1) & x(nL-2) & \cdots & x(nL-N)\\ \vdots & \vdots & \ddots & \vdots \\ x(nL-L+1) & x(nL-L) & \cdots & x(nL-L-N+2) \end{array}\right]$$

where *L* is the length of the block and *N* is the size of the filter. In addition, the desired signal d(n) is described by:

(13) $$d\left(n\right)={\left[d\left(nL\right),\;d\left(nL-1\right),\;\cdots \;d\left(nL-N+1\right)\right]}^{T}$$

We use a convex combination to obtain the output of the filter, as follows:

(14) $$y\left(n\right)=\lambda \left(n\right)\cdot y_{GWO}\left(n\right)+\left[1-\lambda \left(n\right)\right]\cdot y_{PSO}$$

(15) $$y_{GWO}\left(n\right)=X\left(n\right)\cdot w_{1}\left(n\right)$$

(16) $$y_{PSO}\left(n\right)=X\left(n\right)\cdot w_{2}\left(n\right)$$

Here, $y_{GWO}\left(n\right)$ and $y_{PSO}$ are the outputs of the GWO and PSO filters, respectively. λ(n) is a mixing parameter defined in range [0,1], given by:

(17) $$\lambda \left(n\right)=\frac{1}{1+e^{-a(n)}}$$

(18) $$a\left(n+1\right)=a\left(n\right)+\mu_{a}\cdot e\left(1\right)\cdot \left\{e_{PSO}\left(1\right)-e_{GWO}\left(1\right)\right\}\cdot \lambda \left(n\right)\cdot \left[1-\lambda \left(n\right)\right]$$

where $\mu_{a}$ is the step size for an auxiliary parameter (a(n)), which is used to minimize the instantaneous square error of the filters, and is calculated at each iteration to obtain the best characteristics of each filter. Specifically, the adaption rule of a(n) specifies its value inside a symmetric interval [$-a^{+},a^{+}$]. Likewise, the value of a(n) limits the permissible range of λ(n) to [1- $\lambda^{+}$, $\lambda^{+}$]. Finally, the error vector for each filter is expressed as follows:

(19) $$e_{GWO}\left(n\right)=d\left(n\right)-y_{GWO}\left(n\right)$$

(20) $$e_{PSO}\left(n\right)=d\left(n\right)-y_{PSO}\left(n\right)$$

In the next section, we present the proposed convex structure by using the GWO and PSO algorithms.

- *The computation of the coefficients of the filter by using the GWO algorithm*
  The encircling behavior of grey wolves is described in Equation (1). Previously, in [22], the calculation of $\vec{A}$ was proposed as follows:
  (21) $$\vec{A}=2\phi \left(n\right)\cdot {\vec{r}}_{1}-\phi \left(n\right)$$
  (22) $$\phi \left(n\right)=\frac{4}{1+e^{-[e_{GWO}\left(n\right)]}}-2$$
  The search space is determined by the ϕ(n) parameter, which is based on the error signal $e_{GWO}\left(n\right)$. To evaluate each search agent, we use a fitness function defined in terms of the mean square error (MSE). Therefore, the fitness value $f_{k}\left(n\right)$ is expressed as follows:
  (23) $$f_{k}\left(n\right)=\frac{1}{L}\sum_{i=1}^{L}e_{k}^{2}\left(i\right)\;k=1,2,\cdots ,P\left(n\right)$$
  (24) $$P\left(n\right)=\left[\frac{2\cdot (P_{max}-P_{min})}{1+e^{-[e_{GWO}\left(n\right)]}}-\left(P_{max}-P_{min}\right)\right]+P_{min}$$
  Here, the number of search agents, denoted by P(n), is dynamically adjusted based on the instantaneous error, as proposed in [22]. In this context, $P_{max}$ and $P_{min}$ represent the maximum and minimum number of search agents, respectively. Finally, the positions of both the search agents and the prey are updated through Equations (5)–(8), and the vector $w_{1}\left(n\right)$ is set equal to ${\vec{W}}_{\alpha }\left(n\right)$.
- *The computation of the coefficients of the filter by using the PSO algorithm*
  To update the filter coefficients, we use the PSO algorithm. Specifically, we set $w_{2}\left(n\right)=w_{gbest}$. To identify the best position, we choose the particle with the lowest fitness value, $f_{k}\left(n\right)$. Each particle represents a vector of filter coefficients, and we update its position by using the following expressions:
  (25) $$w_{i}\left(n+1\right)=w_{i}\left(n\right)+v_{i}\left(n+1\right)$$
  (26) $$v_{i}\left(n+1\right)=\phi \left(n\right)\cdot v_{i}\left(n\right)+c_{1}\cdot r_{1}\left[w_{pbest}-w_{i}\left(n\right)\right]+c_{2}\cdot r_{2}\left[w_{gbest}-w_{i}\left(n\right)\right]$$
  where $v_{i}\left(n\right)$ is the velocity vector and ϕ(n) is the inertia weight, which is linearly decreased to control exploration and exploitation, given by:
  (27) $$\phi \left(n\right)=\phi_{max}-\frac{n\cdot (\phi_{max}-\phi_{min})}{MaxIter}$$

## 3. Results

**Figure 2:**
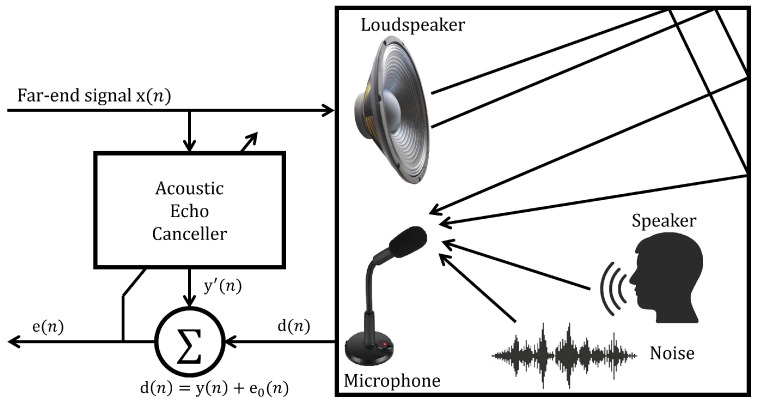
Structure of the acoustic echo canceller.

Here, we simulate the proposed AEC system based on a new convex combination. Specifically, this combination uses the grey wolf optimization (GWO) and particle swarm optimization (PSO) algorithms. Figure 2 shows the experimental setup used to evaluate the performance of the proposed AEC system, where x(n) refers to the original audio signal, e(n) is the residual echo signal, and d(n) is the speech signal combined with the background noise, $e_{0}\left(n\right)$, along with the acoustic echo signal, y(n).

**Figure 3:**
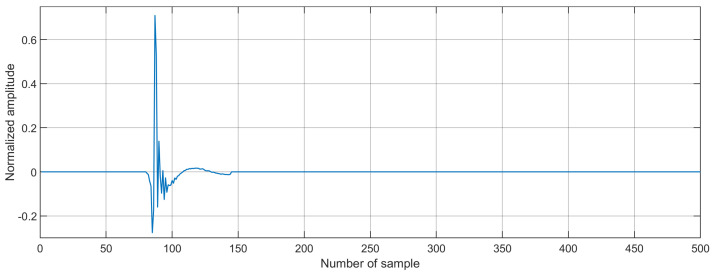
Acoustic echo path used for the simulation of the proposed and existing algorithms.

To perform the simulation of the proposed AEC system, we consider the following conditions:

1. We use an impulse response as the echo path since this response is recommended by ITU-T G168 [26]. In addition, the length of the path is defined by 500 coefficients, as shown in Figure 3.
2. We use the AR(1) process as an input signal. In addition, the echo signal was mixed with white Gaussian noise (SNR = 20 dB).
3. In the case of using the GWO algorithm, the number of search agents is defined in the range of 15–30. On the other hand, the implementation of the PSO algorithm requires up to 60 particles.
4. Here, we test the tracking capabilities of the proposed algorithm by inducing an abrupt change in the impulse response of the acoustic echo path; i.e., we multiply the acoustic paths by −1 in the middle of the adaptive filtering process.
5. The performance of the proposed algorithm is validated in terms of echo return loss enhancement ($ERLE=10log_{10}\left(\frac{d{\left(n\right)}^{2}}{e{\left(n\right)}^{2}}\right)$).
6. The computer performs 2,000,000 iterations to obtain the desired result.

In addition, we define and calibrate all the variables and parameters of the GWO and PSO algorithms to obtain the best performance of the proposed AEC system. In addition, we simulate existing approaches [22,24,25,27,28,29,30,31] to compare the performance between the proposed and existing algorithms. In all cases, we adjust the parameters to obtain the best result of each algorithm.

- *Modified artificial bee colony, MABC*: population size =50, evaporation parameter =0.1, upper bound =1, lower bound =−1, pheromone =0.6, convergence factor $=3\times 10^{-5}$.
- *Artificial bee colony, ABC*: upper bound =1, lower bound =−1, evaporation parameter =0.1, pheromone =0.6, population size =50.
- *Grey wolf optimizer, GWO*: upper bound =1, lower bound =−1, population size =50, *a* decreases linearly from 2 to 0.
- *Particle swarm optimization, PSO*: population size =100, upper bound =1, lower bound =−1, acceleration coefficient, $c_{2}=1$, acceleration coefficient, $c_{1}=1.6$, inertia weight =0.8.
- *PSO-LMS*: population size =60, acceleration coefficient, $c_{2}=1.2$, acceleration coefficient, $c_{1}=0.00005$, convergence factor $=1\times 10^{-9}$, inertia weight =1, upper bound =1, lower bound =−1.
- *Differential evolution, DE*: combination factor =0.25, scaling factor =0.8, crossover rate =0.35, upper bound =1, lower bound =−1, population size =50.
- *Least mean squares, LMS*: convergence factor $=9\times 10^{-7}$.
- *GWO/LMS*: upper bound =1, lower bound =−1, population size automatically adjusted between [30−15], convergence factor $=9\times 10^{-7}$.

**Figure 4:**
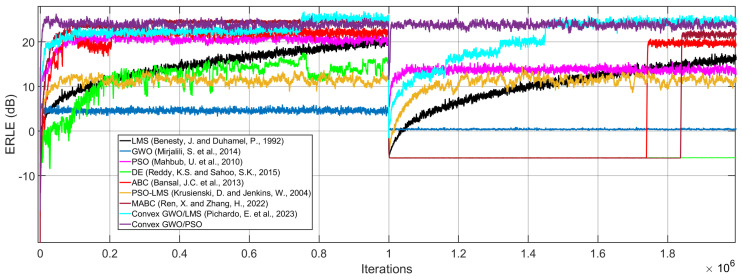
Simulating existing approaches and the proposed algorithm to obtain the ERLE [22,24,25,27,28,29,30,31].

As can be seen in Figure 4, the proposed convex GWO/PSO adaptive filter exhibits the best performance in terms of the ERLE level and convergence speed at the cost of performing more addition and multiplication in comparison with existing approaches, as shown in Table 1. However, the conventional GWO algorithm performs more additions and multiplications and exhibits a very low ERLE level compared with the proposed algorithm. Therefore, our proposal significantly improves the performance of conventional GWO in terms of ERLE and the number of arithmetic operations. In addition, the proposed algorithm exhibits a superior performance compared to existing solutions, especially when the path suffers an abrupt change. This has special relevance, since our approach can effectively reduce acoustic echo signals at high speeds. Therefore, the use of existing approaches in real-world echo noise applications is limited.

To verify the tracking properties of the proposed GWO/PSO algorithm, we performed four different experiments under the following conditions:

- We used an AR(1) process as an input signal.
- We change the SNR from 20 dB to 10 dB in the middle of iterations.
- We induce an abrupt change in the impulse response of the acoustic echo path at the middle of the process. To achieve this, we multiply the acoustic path by −1.
- We shift the acoustic path in the middle of the process.
- We simulate a double-talk scenario in the middle of the process.

**Figure 5:**
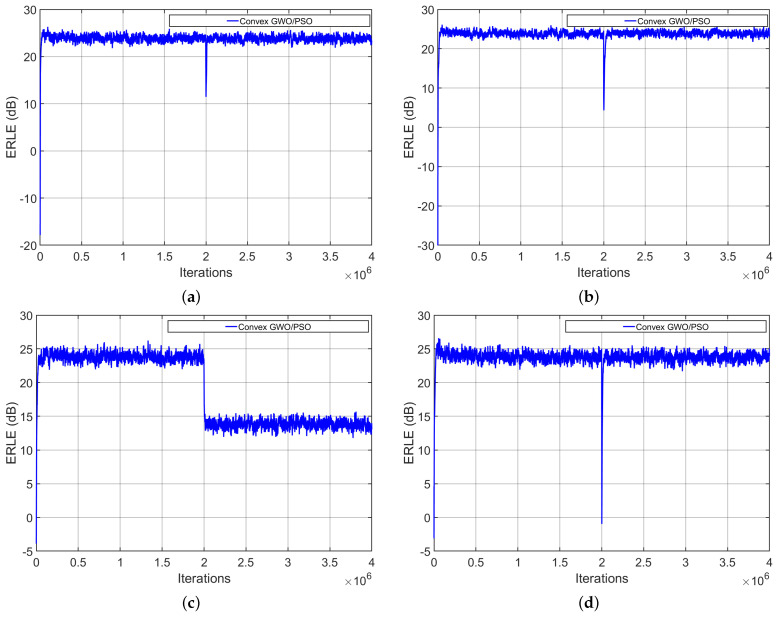
Performing four different experiments to obtain the ERLE curves by using the proposed convex GWO/PSO algorithm. (**a**) Multiplying the acoustic path by −1; (**b**) shifting the acoustic path; (**c**) varying the SNR from 20 to 10 dB; (**d**) considering a double-talk simulation.

Figure 5 shows the performance of the proposed algorithm when considering the previous conditions. In all cases, the algorithm shows a high convergence speed to confirm its tracking capabilities.

In real-world echo noise applications, variations in background noise critically affect the performance of the AEC system. However, our proposal shows good capabilities; the background noise variation does not affect its performance, as shown in Figure 5. To demonstrate this, we decreased the SNR from 20 to 10 dB in the middle of the iterations.

Additionally, we perform three different tests under the following conditions:

- We use the speech signal as an input signal.
- We simulate a single-talk and a double-talk scenario.
- We change the SNR from 20 dB to 10 dB in the middle of the iterations.

**Figure 6:**
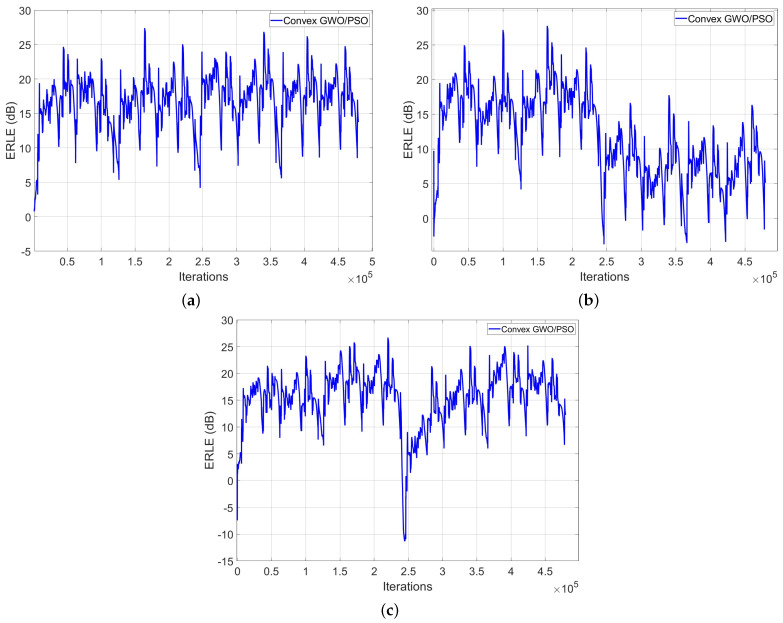
ERLE curves after using the proposed convex GWO/PSO algorithm and considering a speech sequence signal as input signal. (**a**) Considering a single talk simulation; (**b**) varying the SNR from 20 to 10 dB; (**c**) considering a double talk simulation.

As can be observed from Figure 6, the proposed algorithm shows its computational capabilities under a single-talk scenario and a double-talk scenario. Regarding the latter, we employed a double-talk detector circuit to avoid adaptation during periods of simultaneous far and near-end speech. As can be observed from the above figures, the proposed algorithm achieves a good ERLE level and shows excellent tracking capabilities for all combinations of input signal levels, echo paths and echo path changes. With regard to the ERLE level, the improvement in this factor allows us to verify its utility in practical and real-world AEC applications.

### Statistical Comparison

To demonstrate the statistical results in the simulation of each algorithm, we calculate the average of the ERLE value (dB) and the standard deviation, as shown in Table 2. This evaluation considers ten individual experiments, each one with 2,000,000 iterations.

As seen in Table 2, the proposed convex GWO/PSO algorithm presents the highest average value between all approaches and it has the second lowest standard deviation. In contrast, the GWO algorithm, which possesses the lowest standard deviation, also obtains the lowest average value.

## 4. Conclusions

Here, we present for the first time a convex combination of the GWO and PSO algorithms to guarantee a higher convergence rate and a higher level of ERLE compared with gradient descent algorithms and conventional metaheuristic methods. This was proven through several experiments in which the proposed convex GWO/PSO algorithm was tested under different acoustic conditions. Consequently, this algorithm can be seen as a potential tool to be used in real-world acoustic echo cancellation scenarios.

## Figures and Tables

**Table 1:** Comparison between the existing approaches and the proposed convex GWO/LMS system in terms of the number of multiplications and additions.

Algorithm	Multiplications	Additions
LMS [27]	90,002,000,000	90,002,000,000
Convex GWO/LMS [22]	157,634,525,000	130,603,915,000
DE [28]	2,250,000,000,000	4,500,000,000,000
ABC [29]	9,000,900,000,000	11,249,100,000,000
MABC [31]	10,891,200,000,000	13,589,400,000,000
PSO-LMS [30]	13,546,200,000,000	13,590,600,000,000
PSO [25]	22,500,600,000,000	22,500,600,000,000
The proposed convex GWO/PSO	22,568,196,525,000	22,541,157,915,000
GWO [24]	33,750,002,000,000	20,250,002,000,000

**Table 2:** Comparison between the existing approaches and the proposed convex GWO/LMS system in terms of the standard deviation in dB and the average value of ERLE.

Algorithm	Standard Deviation	Average Value
This work	0.7467	23.8965
Convex GWO/LMS [22]	2.0259	22.7590
ABC [29]	3.4164	21.0656
DE [28]	4.4434	11.7972
GWO [24]	0.3892	4.5653
LMS [27]	4.1761	14.7202
MABC [31]	3.0326	23.6927
PSO [25]	1.5622	20.2452
PSO-LMS [30]	1.1497	11.2859

## Data Availability

The raw data supporting the conclusions of this article will be made available by the authors on request.
